# Early prediction of response to radiotherapy and androgen-deprivation therapy in prostate cancer by repeated functional MRI: a preclinical study

**DOI:** 10.1186/1748-717X-6-65

**Published:** 2011-06-08

**Authors:** Kathrine Røe, Manish Kakar, Therese Seierstad, Anne H Ree, Dag R Olsen

**Affiliations:** 1 Department of Radiation Biology, Institute for Cancer Research, The Norwegian Radium Hospital, Oslo University Hospital, PO Box 4953 Nydalen, 0424 Oslo, Norway; 2 Institute of Clinical Medicine, University of Oslo, Oslo, Norway; 3 Department of Oncology, Akershus University Hospital, Lorenskog, Norway; 4 University of Bergen, Bergen, Norway

**Keywords:** artificial neural network, back propagation neural network, diffusion weighted magnetic resonance imaging, dynamic contrast-enhanced magnetic resonance imaging, prostate cancer, androgen-deprivation therapy, radiotherapy

## Abstract

**Background:**

In modern cancer medicine, morphological magnetic resonance imaging (MRI) is routinely used in diagnostics, treatment planning and assessment of therapeutic efficacy. During the past decade, functional imaging techniques like diffusion-weighted (DW) MRI and dynamic contrast-enhanced (DCE) MRI have increasingly been included into imaging protocols, allowing extraction of intratumoral information of underlying vascular, molecular and physiological mechanisms, not available in morphological images. Separately, pre-treatment and early changes in functional parameters obtained from DWMRI and DCEMRI have shown potential in predicting therapy response. We hypothesized that the combination of several functional parameters increased the predictive power.

**Methods:**

We challenged this hypothesis by using an artificial neural network (ANN) approach, exploiting nonlinear relationships between individual variables, which is particularly suitable in treatment response prediction involving complex cancer data. A clinical scenario was elicited by using 32 mice with human prostate carcinoma xenografts receiving combinations of androgen-deprivation therapy and/or radiotherapy. Pre-radiation and on days 1 and 9 following radiation three repeated DWMRI and DCEMRI acquisitions enabled derivation of the apparent diffusion coefficient (ADC) and the vascular biomarker *K*^trans^, which together with tumor volumes and the established biomarker prostate-specific antigen (PSA), were used as inputs to a back propagation neural network, independently and combined, in order to explore their feasibility of predicting individual treatment response measured as 30 days post-RT tumor volumes.

**Results:**

ADC, volumes and PSA as inputs to the model revealed a correlation coefficient of 0.54 (p < 0.001) between predicted and measured treatment response, while *K*^trans^, volumes and PSA gave a correlation coefficient of 0.66 (p < 0.001). The combination of all parameters (ADC, *K*^trans^, volumes, PSA) successfully predicted treatment response with a correlation coefficient of 0.85 (p < 0.001).

**Conclusions:**

We have in a preclinical investigation showed that the combination of early changes in several functional MRI parameters provides additional information about therapy response. If such an approach could be clinically validated, it may become a tool to help identifying non-responding patients early in treatment, allowing these patients to be considered for alternative treatment strategies, and, thus, providing a contribution to the development of individualized cancer therapy.

## Background

Prostate cancer (PCa) is a disease characterized by biologically heterogeneous behaviour. Treatment of PCa is controversial, with no established consensus on screening or diagnostic tests for pre-treatment evaluation of PCa aggressiveness [1,2]. Consequently, the ability to differentiate between low-risk and high-risk patients and the need for and appropriateness of treatment at any stage of the disease, remains a difficult issue. Whereas low-risk PCa patients are faced with problems associated with over-treatment, high-risk PCa patients might be suffering from under-treatment and high frequency of recurrence. Thus, PCa represents a disease in which early prediction of ultimate therapeutic efficacy is critical, but so far has been challenging to achieve.

Prediction of individual therapy response is critically dependent on the ability to quantify tumor heterogeneity and heterogeneous response of tumors with otherwise identical clinical prognostic factors. Established, non-invasive methods that effectively evaluate the heterogeneous therapy responses are elusive in clinical practice. Radiological response to treatment is most commonly quantified by measuring the tumor diameter in one or two directions. However, functional magnetic resonance imaging (MRI) techniques, like diffusion-weighted (DW) MRI and dynamic contrast-enhanced (DCE) MRI, are promising and have opened for repeated *in vivo *assessment of biomarkers from underlying vascular, molecular and physiological processes in individual tumors. DWMRI depicts the local microstructural characteristics of water diffusion, can be quantified by calculating the apparent diffusion coefficient (ADC), and enables detection of microscopic changes in tissue structure and physiology [3,4]. Further, by tracking the entrance of a diffusible contrast agent from the tumor vasculature and into the extravascular, extracellular space, DCEMRI allows deduction of the vascular biomarker *K*^trans ^[5], which may be of particular importance in clinical response monitoring of increased or inhibited angiogenesis. *K*^trans ^has also been shown to reflect tumor oxygenation status, which is an important factor for successful radiotherapy (RT) outcome [6]. Alterations in functional imaging parameters have been shown to precede tumor volume reductions, enabling identification of good and poor responders at an early time-point, and thus, facilitation of individualized treatment schedules [6-13]. These functional MRI techniques are now increasingly becoming in routine use in many radiological departments, thus, the approach presented in the current study suggests a further use of these data, by exploiting them in prediction modeling together with standard clinical parameters.

Medical artificial intelligence is a methodology that potentially can support clinicians in deciding correct diagnosis, making therapeutic decisions and predicting therapeutic outcome [14,15]. Artificial neural networks (ANNs) are attractive analytical tools in medicine due to their ability to learn from historical examples, analyze non-linear data and being able to generalize a model to independent data. ANNs are inspired by the biological nervous system and consist of interconnected processes utilizing parallel computations, analogous to the biological neurons being the brain's processing units. There are numerous ANN methods, however, this study concentrates on the back propagation neural network (BPNN) approach. This method was originally described by Rumelhart *et al *[16], and has become one of the most popular ANN algorithms in medicine, due to the demonstration of high prediction outcomes in a range of medical applications, which also inspired us to implement and test this approach. The BPNN architecture consists of many identical nodes, or neurons, mainly consisting of nonlinear activation functions. The nodes are interconnected by weights, representing the inter-neuron synapses in the brain. Further, the BPNN architecture is divided into three layers; input, hidden and output layers. The input layer feeds information into the network, while the nodes in the hidden layers and output layers process the information. The nodes in the hidden layer do not have predefined initial values, but do allow complex relationships between input and output nodes to develop. The training process consists of forward and backward propagation of signals. In the forward training process, input data are forwardly propagated in the network while known output parameters are kept in the output nodes to compare the results generated by the network. In the back propagation training phase, the respective differences (errors) are used to change the interconnecting weights by using a gradient learning algorithm by back propagating the errors [16].

ANNs have previously been applied on PCa patient data in order to predict treatment outcome based on clinical parameters like tumor volume, prostate-specific antigen (PSA), the primary and regional nodal extent of the tumor and the absence or presence of metastases (TNM classification), biopsy Gleason score and age as input parameters [14,17-21]. In the study by Gulliford *et al *[17], volume, PSA and tumor stage were used as inputs. Although the results from this study unveiled a predictive potential, both the sensitivity and the specificity were low (sensitivity; 66.8 - 70.2%, specificity; 52.7 - 64.2%). Further, Stephan *et al *[21] performed a study where PSA was used as input to an ANN approach in order to investigate whether such a model could differentiate between PCa and benign prostatic disease. Also in this study the specificity was low; median values 62.1% and 45.5%, for 90% and 95% sensitivity, respectively. We hypothesize that the addition of functional magnetic resonance imaging (MRI) parameters into a prediction model might provide valuable intratumoral information that, in addition to the established clinical parameters, will contribute to improve the prediction of therapeutic efficacy.

To explore whether the combined use of pre-treatment and early therapy-induced changes in functional MRI parameters increases the prediction of therapeutic response, we elicited a clinical scenario by using human, androgen-sensitive prostate carcinoma xenografts receiving RT and/or androgen-deprivation therapy (ADT). Functional MRI parameters were derived after three repeated DWMRI and DCEMRI sessions, and together with volumes and PSA measurements, these parameters were independently and combined used as inputs to a BPNN in order to explore their feasibility of predicting treatment response measured as 30 days post-RT tumor volumes.

## Methods

**Figure 1:**
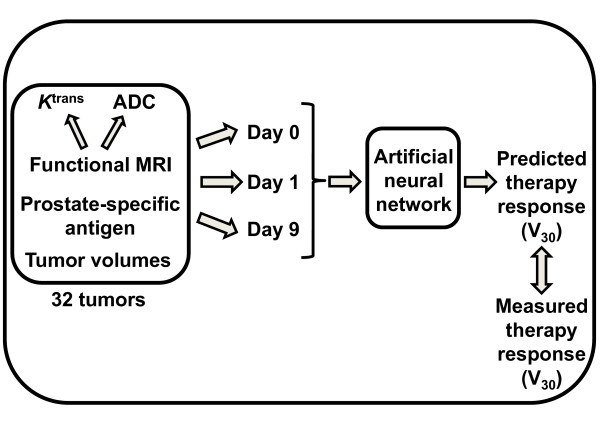
**Schematic synopsis of the study**. Androgen-sensitive prostate carcinoma xenografts received combinations of androgen-deprivation therapy (ADT) and/or radiotherapy (RT) and were subjected functional magnetic resonance imaging (MRI) pre-treatment and 1 and 9 days after onset of treatment. Together with standard clinical parameters (prostate-specific antigen (PSA), tumor volumes) the functional MRI parameters reflecting structural composition (apparent diffusion coefficient (ADC)) and vascularization (*K*^trans^) were used as inputs in an artificial neural network to elucidate how these functional MRI parameters independently and combined affected the prediction of therapy response, as measured by the 30 days post-RT tumor volumes (V_30_).

A schematic synopsis of the experiment is provided in Figure 1.

### Animals, xenografts and treatment

Male, sexually mature BALB/c nude mice (30 - 35 g, 6 - 8 weeks old) were subcutaneously (s.c.) implanted with ~ (2 × 2 × 2) mm^3 ^tumor tissue from the human, androgen-sensitive CWR22 xenograft. Procedures for implantation of xenografts are previously described [22]. All animal experiments were performed according to protocols approved by the animal care and use committee.

Animals were included in the experiment when their shortest tumor diameter reached 8 mm. Androgen-deprived CWR22 xenografts (CWR22-cas) were obtained by surgical castration of animals bearing CWR22 xenografts at a shortest tumor diameter of 13 mm; the animals were included in the experiment when CWR22-cas xenografts had regressed to a shortest diameter of 8 mm. The time from castration to inclusion was 36 ± 4 days. Totally 32 animals (4 groups of 8 animals) were used; CWR22 control, CWR22 irradiation, CWR22-cas control and CWR22-cas irradiation. At inclusion, animals were subjected to a pre-treatment (day 0) MRI before tumors in the irradiation groups received a single-dose of 15 Gy from a ^60^Co source (Mobaltron 80, TEM Instruments, Crawley, UK) with a dose rate of 0.8 Gy/min. At day 1 and day 9 repeated MRIs were performed of all animals.

Anesthesia was provided as s.c. injections of a mixture of 2.4 mg/ml tiletamine and 2.4 mg/ml zolazepam (Zoletil vet, Virbac Laboratories, Carros, France), 3.8 mg/ml xylazine (Narcoxyl vet, Roche, Basel, Switzerland), and 0.1 mg/ml butorphanol (Torbugesic, Fort Dodge Laboratories, Fort Dodge, IA), diluted 1:5 in sterile water. A dose of 50 μl/10 g was given prior to irradiation and 75 μl/10 g before MRI and castration. Castrated animals received analgesia as 0.1 mg/kg s.c. injections of buprenorphine (Temgesic; Schering-Plough, Brussels, Belgium).

### MRI acquisition and analysis

MRI was acquired at day 0 (pre-RT), day 1 and day 9, using a 1.5 T GE Signal LS scanner (GE Medical Systems, Milwaukee, WI). Animals were imaged using an MRI mouse coil [23], while the temperature was maintained at 38°C. First, the tumor was localized using axial fast spin-echo (FSE) T2-weighted (T2W) images (echo time (TE_eff_) = 85 ms, repetition time (TR) = 4000 ms, echo train length (ETL) = 16, image matrix (IM) = 256 × 256, field-of-view (FOV) = 4 cm, slice thickness (ST) = 2 mm). Second, diffusion-weighted images (single shot FSE; TE_eff _= 78.8 ms; TR = 5000 ms; FOV = 14 cm; IM = 128 × 128; ST = 2 mm; interslice gap = 1 mm; b-values = 0 and 100 s/mm^2^) were acquired with the following x, y, and z directions; [1 0 1], [-1 0 1], [0 1 1], [0 1 -1], [1 1 0] and [-1 1 0]. An axial FSE T2W sequence with identical FOV as the DWMRI was obtained for post-processing image analysis purposes. Third, the DCEMRI acquisitions were obtained as described elsewhere [22]. Briefly, a catheter attached to a cannula with saline-diluted Gd-DTPA (Magnevist^®^, Schering, Berlin, Germany) was inserted into the tail vein. Dynamic T1-weighted (T1W) imaging was acquired by performing 20 minutes of dynamic fast spoiled gradient-recalled (FSPGR) imaging after the initial 5 pre-contrast images and the 3 seconds injection of the contrast agent. Time resolution was 12 seconds and the voxel size was 0.23 × 0.47 × 2 mm^3^. Proton density images were acquired prior to and after DCEMRI to allow quantification of the concentration of Gd-DTPA [24]. The vascular input function (VIF) needed in quantitative post-processing image analysis was VIF = 3.57 ± 0.34 mM (exp((-0.025 ± 0.005 s^-1^)t)) + 1.45 ± 0.15 mM (exp((-0.0074 ± 0.0036 s^-1^)t)) (22).

Post-processing DWMRI analysis was performed in nICE (Nordic NeuroLab, Bergen, Norway). Isotropic ADC maps were calculated voxel-wise using a mono-exponential approach, allowing determination of mean tumor ADCs after transferring tumor region-of-interests (ROIs) delineated in T2W MR images to the ADC maps. DCEMRI analysis was executed in IDL (Interactive Data Language v6.2, Research Systems Inc., Boulder, CO). ROIs were traced in post-contrast T1W images, before contrast enhancement curves from individual voxels were fitted to the kinetic model of Tofts [5], allowing voxel-wise and mean tumor estimation of the vascular biomarker *K*^trans ^(s^-1^).

### PSA

Blood samples from all animals at days 0, 1 and 9 were obtained and allowed to coagulate before being centrifuged and stored at -80°C until analysis. Free and total PSA were assayed by the fluoroimmunonometric AutoDELFIA ProStatus™ PSA Free/Total kit (PerkinElmer Life and Analytical Sciences, Wallac Oy, Turku, Finland).

### Treatment response monitoring

From the day of implantation until day 30 post-irradiation, tumor volumes were estimated from caliper measurements by using the formula *(length × length × width)/2*, with *length *being the longest diameter across the tumor and *width *the corresponding perpendicular.

### ANN simulations

**Figure 2:**
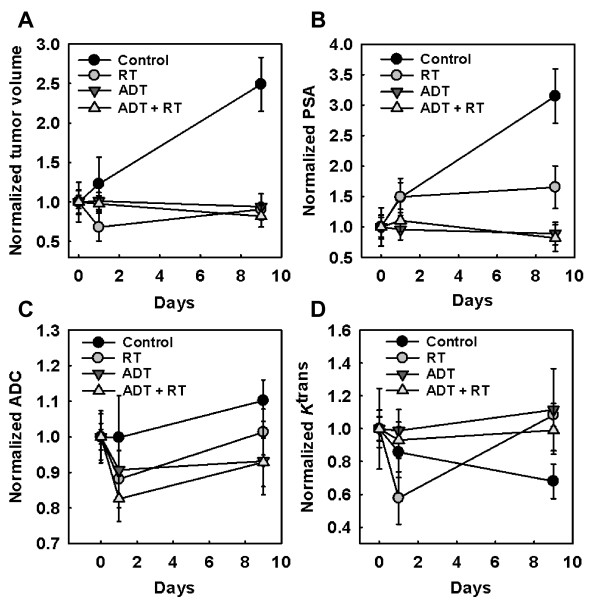
**Input parameters to the artificial neural network model**. Tumor volumes (V), prostate-specific antigen (PSA), the apparent diffusion coefficients (ADC) and the vascular biomarker *K*^trans ^were acquired pre-treatment and early in treatment course and normalized to the baseline (day 0) measurement. These parameters were used as inputs to the back propagation neural network (BPNN) in order to explore whether they could predict therapeutic outcome, as measured by the 30 days post-radiotherapy (RT) tumor volumes.

Tumor volumes (V), PSA, ADC and *K*^trans ^acquired pre-treatment and early in treatment course were normalized to the individual baseline (day 0) measurement (Figure 2) and used as inputs to a BPNN to explore whether these parameters could predict treatment response, as measured by individual tumor volumes 30 days post-irradiation (V_30_). Additionally, four categorical (binary encoded) variables representing treatment groups were used in all simulations. The BPNN repeatedly adjusted the weights of the network and the threshold of each neuron according to a criterion that the cost function minimized. The cost function was a root mean squared error (RMSE) between the target outputs and the actual outputs of the network. The two steps of the learning process included:

a) *Forward propagation*. The value of the calculated output, *y_j_*, was compared to the actual output, *O_j_*, before the output differences were inserted into the error function *E *defined as:

where *M *is the total number of tumor response patterns given as input to the network, *N *is the total number of output nodes of the network, and *j *a specific output node, given a specific pattern *i *into the network.

b) *Backward propagation*. The error *E *from equation above was back propagated by updating the weights, *w_ij_*, using scaled conjugate gradient descents:

where *η *(0 <*η *< 1) controlled the learning rate of the algorithm. The learning process continued until *E *converged to a predefined value or until the maximum number of epochs was reached. An epoch is a single pass of the data through the network, i.e. the different tumor response patterns (V, PSA, ADC and/or *K*^trans^) for all experimental groups through the training set, followed by the validation set and the testing set.

All ANN simulations were performed in the Matlab Neural Network Toolbox, software version 4.0.2 (The Mathworks, Inc., Natick, MA).

**Figure 3:**
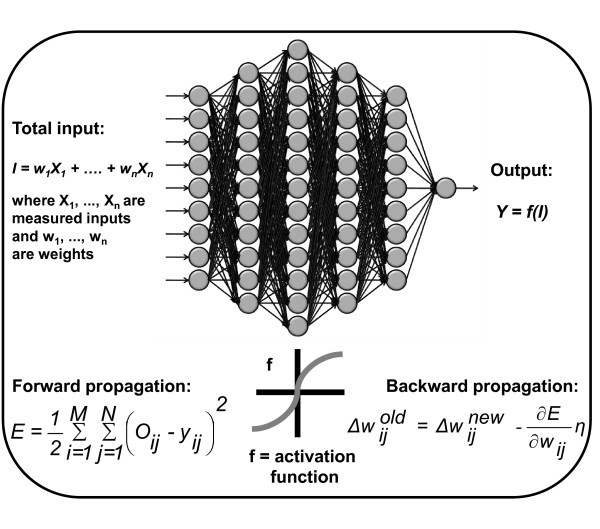
**Illustration of the architecture of the back propagation neural network (BPNN)**. The BPNN repeatedly adjusts the weights, *w*, of the network and the threshold of each neuron (grey circles). The two steps of the learning process include forward propagation, where the predicted output value, *O*, is compared to the actual output value, *y*, and backward propagation, where the error, *E*, from this comparison is back propagated by updating the weights using a scaled conjugated gradient descent algorithm. The *η *(0 <*η *< 1) is a constant controlling the convergence rate of the algorithm. A six-layered network approach and a sequential mode for training were used in all simulations, keeping *η *= 0.4.

Three different simulations with different sets of input parameters were performed using the same BPNN architecture. For all simulations, the BPNN used six hidden layers and a sequential mode for training, while keeping *η *= 0.4. The first simulation used a dataset consisting of numerical normalized inputs of ADC, V and PSA from days 0, 1 and 9, and categorical variables representing treatment groups. The architecture, including hidden layers, of the neural network is illustrated in Figure 3. In the second simulation, the dataset consisted of numerical *K*^trans^, V and PSA values from days 0, 1 and 9, in addition to the treatment groups. The last simulation included all numerical parameters (ADC, *K*^trans^, V and PSA) and treatment groups.

### Statistical analysis

Using a significance level of 5%, the Pearson's correlation test (SPSS 16.0, SPSS, Cary, NC) assessed whether correlations between variables were significant.

## Results

Ultimate treatment response was measured as tumor volumes at day 30 (V_30_). Volumes of tumors in the untreated group increased with 940 ± 91% compared to baseline (day 0) volumes, whereas tumors receiving radiation were 60 ± 25% larger at the endpoint than at baseline. Androgen-deprivation alone resulted in reduction in tumor volumes by 40 ± 9% compared to baseline, whereas tumors receiving combined androgen-deprivation and radiotherapy presented a 64 ± 5% tumor volume reduction at the experimental endpoint.

**Figure 4:**
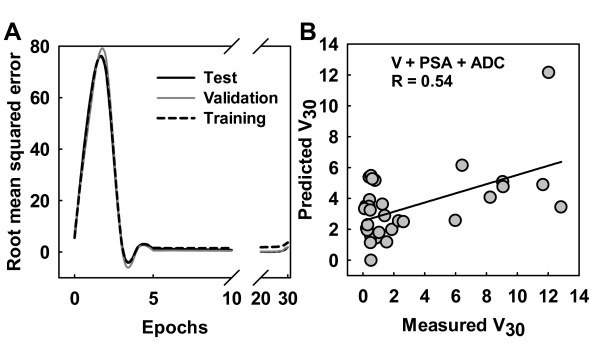
**Prediction of treatment response using apparent diffusion coefficients from diffusion-weighted MRI**. The root mean square error (RMSE) plot visualizes the performance of the training, validation and test data (A). By using apparent diffusion coefficients (ADC), tumor volumes (V), prostate-specific antigen (PSA) and treatment groups as inputs to the back propagation neural network (BPNN) a correlation coefficient of 0.54 (p < 0.001) was found between predicted and measured treatment response (V_30_) (B).

By using a BPNN with a scaled conjugate gradient learning algorithm, 50% of the data were used for training, 25% for validation and 25% for testing. The RMSE plot in Figure 4A shows the performance of the testing and validation of the first simulation, where normalized values of ADC, PSA and tumor volumes from days 0, 1 and 9, in addition to treatment groups (binary encoded), were used as input variables. For this simulation, the RMSE increased after 30 epochs, indicating overtraining. Thus, the optimal training for the network was found to be 30 epochs. The optimal number of epochs for the second and third simulations was decided by a similar approach as above, and found to be 49 and 69, respectively.

**Figure 5:**
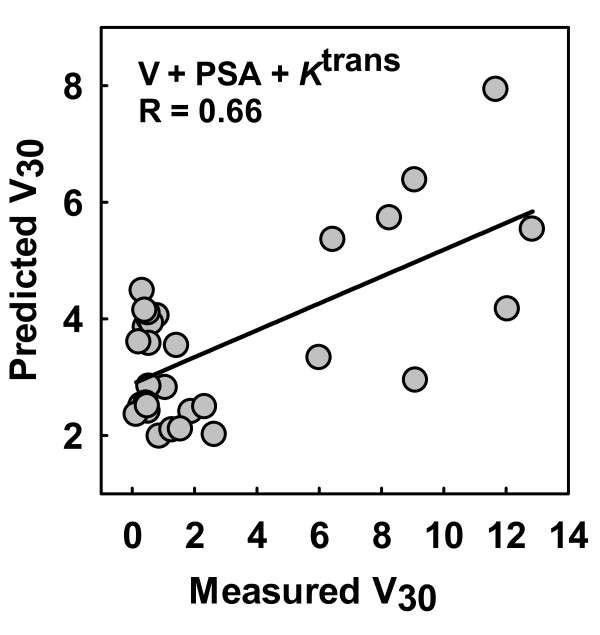
**Prediction of treatment response using *K*^trans ^from dynamic contrast-enhanced MRI**. By using *K*^trans^, tumor volumes (V), prostate-specific antigen (PSA) and treatment groups as inputs to the back propagation neural network (BPNN) a correlation coefficient of 0.66 (p < 0.001) was found between predicted and measured treatment response (V_30_).

**Figure 6:**
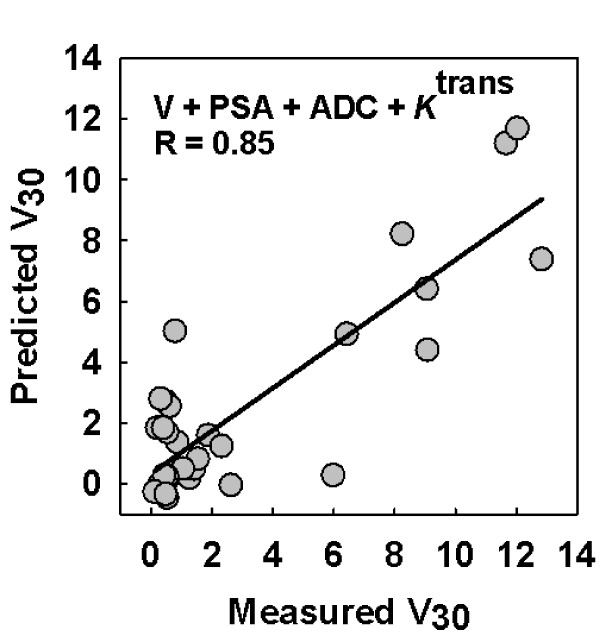
**Combining multiple functional MRI parameters improves prediction of treatment response**. By combining all parameters (tumor volumes (V), prostate-specific antigen (PSA), apparent diffusion coefficients (ADC), *K*^trans^) and treatment groups, the treatment response was predicted with a correlation coefficient of 0.85 (p < 0.001) between predicted and measured V_30_.

The use of ADC together with tumor volumes, PSA and treatment groups as inputs to the BPNN model revealed a correlation coefficient of 0.54 (p < 0.001) between predicted and measured treatment response (V_30_) (Figure 4B). By replacing the ADC with *K*^trans^, the correlation coefficient increased to 0.66 (p < 0.001) (Figure 5). However, the combination of all parameters (V, PSA, ADC, *K*^trans^) predicted treatment response with a correlation coefficient of 0.85 (p < 0.001) between predicted and measured V_30 _(Figure 6). This approach was superior to all other ANN simulations using the parameters independently.

## Discussion

Assessment of therapeutic efficacy in PCa patients represents a controversial issue in clinical medicine due to the heterogeneity of the disease and its unpredictable treatment response. Although the use of ADT and/or RT causes tumor regression, complete remission often fails and high-risk patients usually present recurrent disease within few years. Classic prognostic factors, like tumor volume, PSA, TNM classification and Gleason score, are those that currently guide therapy selection. However, these may be suboptimal in predicting outcome for individual patients, as these factors are not accounting for the underlying heterogeneity in vascular, molecular and physiological processes causing large variations in individual tumor responses. Thus, these factors may not enable prediction of treatment failure early in the course of treatment, when therapeutic adjustments still are feasible in terms of e.g. radiation dose escalation or alterations in concurrent therapy.

The benefits of ANNs compared to conventional regression statistics comprise the capability of being more accurate for large and complex data materials, e.g. patient data with multiple parameters from multiple measurement time-points. The artificial intelligence models of biological systems can be generated without needing assumptions about the underlying statistical distributions. Currently, *in vivo *imaging techniques are rapidly evolving and being extensively tested for their capability of correctly reflecting biological and physiological properties of tumor tissue. Such functional information is particularly beneficial for ANNs, since data from multiple sources effectively can be incorporated without needing knowledge on the combination of underlying biological information.

The presented results were obtained in a preclinical study in prostate cancer xenografts, and suggest that the combination of functional MRI parameters, in addition to standard clinical parameters, increases the power of predicting therapeutic outcome in prostate carcinoma after treatment with ADT and/or RT. Our two first simulations included each individual tumor's ADC from DWMRI, or *K*^trans ^from DCEMRI, respectively, in addition to the standard clinical parameters tumor volume and PSA. The correlations between the BPNN predicted and the measured treatment response were found to be significant, but not very strong (R = 0.54 and R = 0.66, respectively). When we combined both the ADC and the *K*^trans ^results in the third simulation, this gave a considerable increase in the correlation between the predicted and measured outcome (R = 0.85), indicating that these parameters together reflect important treatment response-related information of the tumors.

Our results were obtained in a human xenograft model, thus, the next step could be to apply the same approach in a clinical setting, including parameters from functional MRI, as well as standard clinical parameters, from PCa patients receiving ADT and/or RT treatment. In the present study, the post-treatment imaging was performed at day 1 and day 9, and these time-points are maybe not easily translated into clinical assessment of early treatment response. However, in recent years, the use of imaging modalities for early-in-treatment response assessments, for example 3 to 8 weeks after initiation of therapy, has increased, and showed potential to evaluate whether the patient respond to the chosen treatment or not. If this could be reliably measured, or predicted, from this early imaging assessment, this may help deciding whether the patient should receive intensified, or altered treatment, or possibly a reduction in unnecessary treatment. Moreover, if accounting for the five times faster metabolism in mice, 9 days would translate into approximately 6 weeks in a human, and thus, this may be a relevant time-point for treatment response evaluation, although not directly comparable. However, the results from the current study suggest a promising additional utilization of the large amounts of image data presently being acquired in hospitals. If the model is validated in clinical data, the presented methodology might become an early assay for treatment response prediction, wherein different pre-treatment and early in-treatment functional imaging parameters may be combined with standard clinical parameters in order to increase the prediction of how individual tumors respond to therapy.

Although all simulations demonstrate significant correlations between the predicted tumor volume 30 days post-RT and the measured tumor volumes, Figures 4B, 5 and 6 also indicate a spread in the data points. This implies that there is a probability of misclassifying the response from individual tumors, meaning that precaution should be taken if extrapolations to individuals are performed uncritically. Further, when using a BPNN, care should also be taken when training the network, in order not to under- or overtrain it [25]. Since our RMSE function (Figure 4A) from training, testing and validating the network showed curve flattening after a few training epochs, this indicated that no overtraining occurred. However, if such a model is to be applied on clinical data, the model must be rigorously validated, for example with respect to the number of layers and epochs. Patient materials will always present a larger biological heterogeneity than xenografts grown in immune-deficient mice, representing a risk for overtraining the network if the BPNN parameters are not chosen cautiously.

## Conclusion

The presented results, derived from a preclinical study in prostate cancer xenografts, indicate that the combination of several functional MRI parameters obtained pre-treatment and early in the course of treatment, into an artificial neural network model, may provide additional, useful information about therapy response. If clinically established, this approach may help identifying non-responding patients early during treatment course, allowing these patients to be considered for alternative treatment strategies, and, thus, providing a contribution to the development of personalized prostate cancer therapy.

## List of abbreviations

ADC: apparent diffusion coefficient; ADT: androgen-deprivation therapy; ANN: artificial neural network; BPNN: back propagation neural network; DCEMRI: dynamic contrast-enhanced magnetic resonance imaging; DWMRI: diffusion-weighted magnetic resonance imaging; PCa: prostate cancer; PSA: prostate specific antigen; RMSE: root mean square error; RT: radiotherapy; V: volume

## Competing interests

The authors declare that they have no competing interests.

## Authors' contributions

KR designed the study, carried out the animal experiments, MRI data acquisition and analysis, participated in ANN simulations and wrote the manuscript. MK developed software and performed the ANN simulations, and contributed in revision of the manuscript. TS and AHR helped in discussion of the data and revision of the manuscript. DRO participated in study design, data discussion and revision of the manuscript. All authors read and approved the final manuscript.
